# Detection of Blood CO_2_ Influences on Cerebral Hemodynamics Using Transfer Entropy

**DOI:** 10.3390/e26010023

**Published:** 2023-12-25

**Authors:** Juan Fernández-Muñoz, Victoria J. Haunton, Ronney B. Panerai, José Luis Jara

**Affiliations:** 1 Departamento de Ingeniería Informática, Facultad de Ingeniería, Universidad de Santiago de Chile, Santiago 9170022, Chile; juan.fernandez.m@usach.cl; 2 Department of Cardiovascular Sciences, University of Leicester, Leicester LE1 7RH, UK; vjh12@leicester.ac.uk (V.J.H.); rp9@leicester.ac.uk (R.B.P.); 3 National Institute for Health Research (NIHR) Leicester Biomedical Research Centre, University of Leicester, Leicester LE5 4PW, UK; 4 British Heart Foundation Cardiovascular Research Centre, Glenfield Hospital, Leicester LE5 4PW, UK

**Keywords:** conditional transfer entropy, information theory, cerebral hemodynamics

## Abstract

Cerebral hemodynamics describes an important physiological system affected by components such as blood pressure, CO_2_ levels, and endothelial factors. Recently, novel techniques have emerged to analyse cerebral hemodynamics based on the calculation of entropies, which quantifies or describes changes in the complexity of this system when it is affected by a pathological or physiological influence. One recently described measure is transfer entropy, which allows for the determination of causality between the various components of a system in terms of their flow of information, and has shown positive results in the multivariate analysis of physiological signals. This study aims to determine whether conditional transfer entropy reflects the causality in terms of entropy generated by hypocapnia on cerebral hemodynamics. To achieve this, non-invasive signals from 28 healthy individuals who undertook a hyperventilation maneuver were analyzed using conditional transfer entropy to assess the variation in the relevance of CO_2_ levels on cerebral blood velocity. By employing a specific method to discretize the signals, it was possible to differentiate the influence of CO_2_ levels during the hyperventilation phase (22.0% and 20.3% increase for the left and right hemispheres, respectively) compared to normal breathing, which remained higher during the recovery phase (15.3% and 15.2% increase, respectively).

## 1. Introduction

Cerebral hemodynamics refers to the regulation of cerebral blood flow and the mechanisms involved in maintaining it within normal ranges [1]. Several factors, including changes in blood pressure, blood carbon dioxide (CO_2_) levels, and neurovascular pathologies, can significantly impact cerebral hemodynamics.

Currently, there are several methods available for studying physiological systems, with entropy-based methods being prominent among them [2]. These methods enable the characterization of systems in terms of complexity, disorder, randomness, and other factors. Many entropy-based calculations are univariate, which often limits the ability to consider all relevant components. Additionally, certain multivariate methods, such as mutual information, may fail to consider the dynamism and directionality of physiological systems [3]. In light of these limitations, a measure of entropy called transfer entropy has emerged. Transfer entropy aims to determine the causality present in a system by assessing the information or entropy of its components, while considering both the dynamism and temporal aspects of these components [3]. It is also known that transfer entropy is equivalent to Granger causality under Gaussian variables [4].

Transfer entropy has demonstrated promising results in the analysis of physiological systems. For example, it has been instrumental in determining the influence of CO_2_ and O_2_ effects on respiratory flow in lambs consuming domperidone [5]. Additionally, it has been used to investigate the relationship between changes in the interaction between the sympathetic and parasympathetic nervous systems and the presence of congestive heart failure in individuals [6]. Transfer entropy has also been utilized to identify topological differences between the brain networks of healthy individuals and those with schizophrenia. Notably, individuals with schizophrenia often exhibit deteriorated or weakened interactions within the network [7]. These examples illustrate the wide array of applications for transfer entropy in physiology research. Transfer entropy is considered a valid alternative for investigating cerebral hemodynamics because of its multivariate approach, which enables the determination of the influence of various variables on its regulatory mechanisms.

It has been suggested that previous studies evaluating the influence of CO_2_ on cerebral blood flow may have overestimated the impact of CO_2_ variations, as they usually attribute all observed changes solely to changes in CO_2_, ignoring the contribution made by concurrent changes in arterial blood pressure, which are also induced by variations in CO_2_ [8]. Transfer entropy presents an opportunity to estimate the influence of CO_2_ on blood flow while eliminating the confounding effect of arterial blood pressure.

The objective of this study is to investigate whether transfer entropy enables the differentiation of the influence of blood CO_2_ levels on cerebral blood velocity during a respiratory exercise in a group of healthy individuals.

## 2. Materials and Methods

### 2.1. Subjects and Signals

The data used in this study were selected from a set of biomedical signals utilized in previous works [9,10]. A total of 28 healthy individuals participated in the study and their data were collected while they were positioned in a supine position on a couch. Continuous recordings of heart rate, arterial blood pressure (BP), end-tidal carbon dioxide (ETCO_2_), and bilateral blood velocity in the middle cerebral arteries (left and right MCAv) were acquired. The heart rate was measured using a 3-lead echocardiography system, BP was monitored using a finger arterial clamping device, ETCO_2_ levels were measured with a nasal capnography module, and MCAv was measured using transcranial Doppler with 2-MHz probes. The collected signals were preprocessed offline and synchronized using the electrocardiography trace. Mean values of BP and MCAv were calculated for each cardiac cycle. Synchronized ETCO_2_ and mean signals were sampled at a fixed rate of 5 Hz for analysis. Ethical approval was obtained from the Northampton Research Ethics Committee, UK (reference 11/EM/0369), and all participants provided written informed consent. For more detailed information about the protocol used, please refer to [9].

All participants underwent a 3-phase respiratory exercise during which their signals were monitored. During the first phase (rest), lasting 60 s, individuals were in a resting state breathing normal room air. In the second phase (hyperventilation), which lasted 90 s, participants synchronized their breathing with an electronic metronome which gradually increased their respiratory rate to 25 breaths per minute. The third and final phase (recovery) lasted for an additional 120 s, during which participants were at rest and resumed normal breathing.

### 2.2. Transfer Entropy

Transfer entropy serves as an extension of Shannon entropy and mutual information, enabling the consideration of dynamic, temporal, and directional characteristics within a given system and its component interactions [3,11]. When given an input process *X* and an output process *Y* (both discrete random variables), transfer entropy quantifies the reduction in uncertainty observed in future values of *Y* by knowing the past values of *X*, considering the past values of *Y* [11] (p. 68). This quantification represents the information transferred from *X* to *Y*.

The transfer entropy between time series *X* and *Y* is mathematically represented (when considering one time lag only) by (1), where *t* denotes a specific point in the respective series at a given time, and *k* and *l* indicate the number of past values to consider [5]. It is important to note that before applying this approach, the time series needs to be transformed into a probability distribution function.

(1) $$T_{X\to Y}=\sum_{y_{t},y_{t-1},x_{t-1}}p\left(y_{t},y_{t-1}^{\left(k\right)},x_{t-1}^{\left(l\right)}\right)\log\frac{p(y_{t}|y_{t-1}^{\left(k\right)},x_{t-1}^{\left(l\right)})}{p(y_{t}|y_{t-1}^{\left(k\right)})}$$

This measure can also be expressed as a function of conditional entropies. By setting the block lengths of past values in *X* and *Y* to one (i.e., k=l=1 ), the expression in (2) can be obtained.

(2) $$T_{X\to Y}=H\left(y_{t}|y_{t-1}\right)-H\left(y_{t}|y_{t-1},x_{t-1}\right)$$

If the transfer entropy $T_{X\to Y}=0$ , it indicates that the history of *Y* is independent of the past of *X*.

In a system, multiple variables may interplay with each other, directly influencing their behaviors. Therefore, it is essential to consider the influence of all variables that interact with each other. However, the transfer entropy from *X* to *Y* alone may not detect synergy caused by additional variables, potentially leading to redundant or spurious results [12]. One option is to modify the transfer entropy by incorporating the influence of additional variables conditionally into (1).

This gives rise to the conditional transfer entropy, as shown in (3). In this case, the conditional part of a third variable *Z*, which directionally influences variable *Y*, is added, similar to variable *X*. This form allows for considering cases where the flow of information develops as X→Z→Y and Z→X∪Z→Y∪X→Y [11].

(3) $$T_{X\to Y|Z}=H\left(Y_{t}|Y_{t-1},Z_{t-1}\right)-H\left(Y_{t}|Y_{t-1},X_{t-1},Z_{t-1}\right)$$

This measure aims to quantify the reduction in uncertainty observed in future values of *Y* by knowing the past values of *X*, *Y*, and *X* conditioned on the entire past of *Z* [13]. Effectively, this rules out the information shared between *X* and *Y* that is mediated by their common interaction with *Z* [14]. Moreover, the synergistic and redundant influence of other variables can be discounted with conditional transfer entropy [13] (p. 76).

To facilitate the comparison of transfer entropy results across different cases, it is necessary to normalize the obtained values for better interpretation. The normalization method used involves estimating the transfer entropy value $T_{X\to Y}$ by averaging the transfer entropy calculated on a set of input signals that have the same probability distribution as the original signal *X*. To achieve this, the probability distribution of *X* is shuffled *n* times, and for each new shuffled signal $X^{\left(s\right)}$ , the transfer entropy is computed using the original signal *Y*. Finally, the obtained values are averaged to obtain an estimated value of transfer entropy, $E{[}^{n}{\hat{T}}_{X^{\left(s\right)}\to Y}]$ . This estimated value is then subtracted from the original transfer entropy, and the result is divided by the entropy rate of *Y*, as shown in (4) [15].

(4) $${\;}^{n}T_{X\to Y}=\frac{T_{X\to Y}-E{[}^{n}{\hat{T}}_{X^{\left(s\right)}\to Y}]}{H(Y_{t}|Y_{t-1})}$$

This normalization process generates a value between 0 and 1, indicating the fraction of information in *Y* that is not explained by its past but rather by *X*. It is worth noting that this normalization is widely used in studies that utilize transfer entropy as a method [16,17,18,19].

### 2.3. Time Series Discretisation

As outlined earlier, to calculate transfer entropy based on discrete random variables, it is necessary to discretize the time series in analysis. This involves transforming the continuous values of these series into discrete or categorical variables.

There are numerous methods for achieving this discretisation, each with advantages and disadvantages [5,11,20]. For this study, it was preferred to use two methods that are simple to compute (complexity proportional to the length of the series) and which do not require complex parameterisations, namely discretisation based on fixed bins and equal frequency bins.

Fixed bin discretization consists of splitting the range of observed values in the time series into *n* equal-size intervals, where *n* is a user-supplied parameter [5]. Discretization by equal frequency bins involves dividing the range of observed values in the time series into *n* intervals, each containing a comparable number of data points. This is achieved by sorting all values in ascending order and then distributing them into *n* disjoint sets, each containing approximately the same number of elements [20].

**Figure 1:**
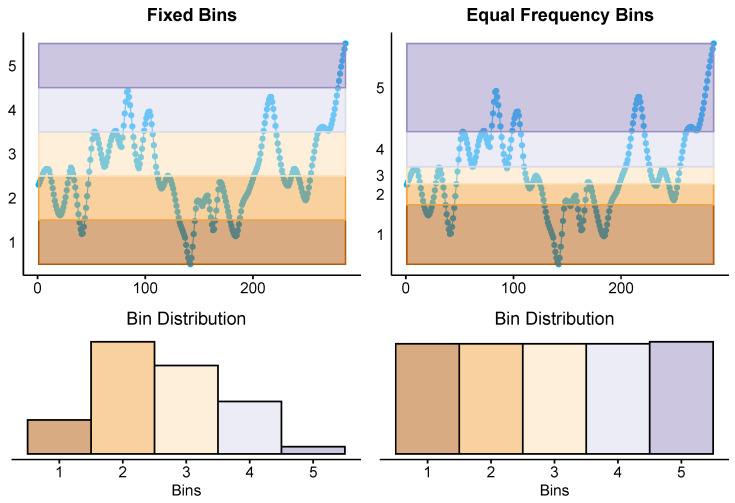
Example of the discretization of an MCAv signal from a healthy subject into five bins using fixed bins (**top left**) and equal frequency bins (**top right**). The discretized sequence is dominated by central bins in the case of fixed bins (**bottom left**), whereas all bins exhibit a similar occurrence with equal frequency bins (**bottom right**).

Figure 1 illustrates the discretization of a sample time series by applying these methods with five bins ( n=5 ). In both cases, the resulting bins are numbered from 1 to *n*. Subsequently, the series is discretized by ”replacing” each data point with the corresponding bin number. It is evident that the central bins appear more frequently in the discrete sequence when using fixed bins. In contrast, as expected, all bins show a similar occurrence when equal frequency bins are applied.

**Figure 2:**
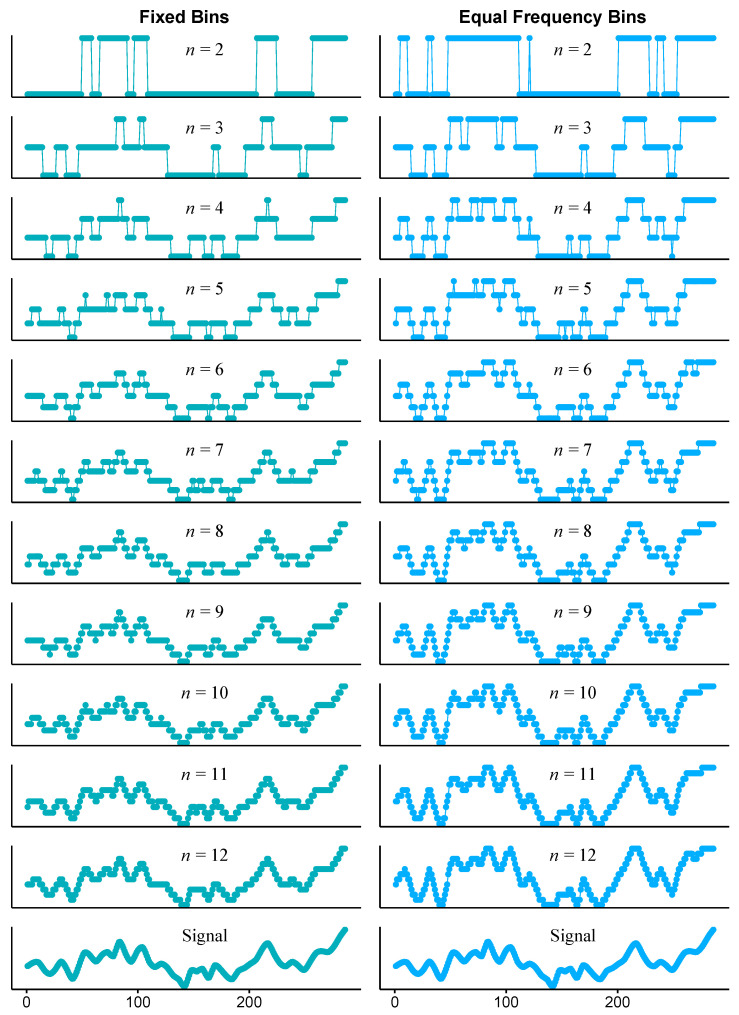
Comparison of the original MCAv signal from a healthy subject (bottom) with its discretized versions using fixed bins (**left**) and equal frequency bins (**right**). The number of bins ranges from 2 to 12 (from top to bottom).

For this study, each hemodynamic recording was discretized using the two methods described above to obtain probability distributions with 12 bins. The decision to employ this number of bins is grounded in considerations of potential information loss resulting from using a smaller number of symbols to represent the volunteers’ signals. Figure 2 illustrate the effect of the number of bins employed for the discretization of the sample signal used in Figure 1. It can be observed that the discrete signals progressively resemble the original one as the number of bins increases. However, higher numbers of bins begin to require much more computation time and memory, as the number of potential patterns to consider grows exponentially.

### 2.4. Procedure

The respiratory exercise performed by the volunteers enabled the registration of cerebral hemodynamic signals during poikilocapnic (Phase 1, Rest) conditions. Previous analyses have indicated that the mechanisms governing BP and MCAv responses to CO_2_ exhibit dynamic characteristics, and that the association between CO_2_ and MCAv is nonlinear, with effects of changes in BP induced by CO_2_ [8].

MCAv signals from each hemisphere were analyzed separately in each of the three phases of the respiratory exercise. After discretization, conditional transfer entropy was calculated between the ETCO_2_ signal, as input, and MCAv, as output, thus eliminating BP contribution, using Equation (3) and normalized according to (4).

Subject mean conditional transfer entropy values were compared using linear mixed-effects models to identify significant differences across respiratory phases used as predictors. A Tukey post hoc procedure was conducted to determine specific differences. A significance threshold of p<0.05 was applied.

## 3. Results

**Figure 3:**
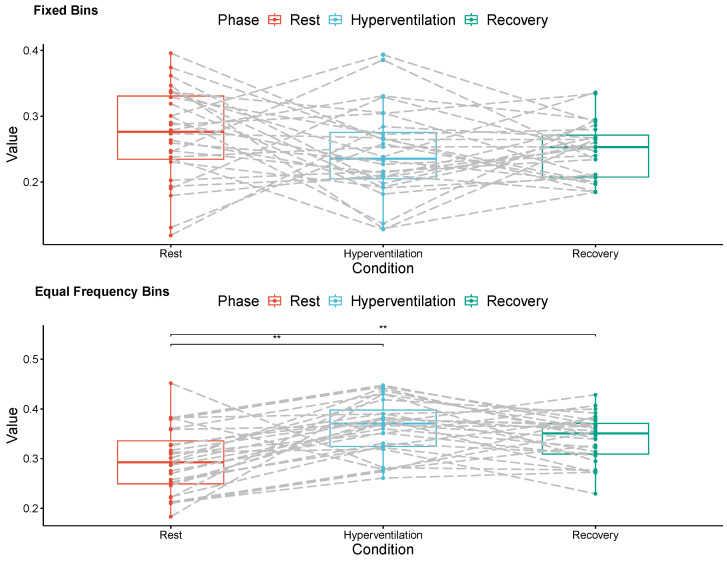
Mean conditional transfer entropy values for all volunteers, estimated from left hemisphere MCAv signals recorded during three respiratory phases (Rest, Hyperventilation, Recovery). The signals were discretized into 12 symbols using fixed bins (**top**) and equal frequency bins (**bottom**). The symbol ** indicates a significant difference.

**Figure 4:**
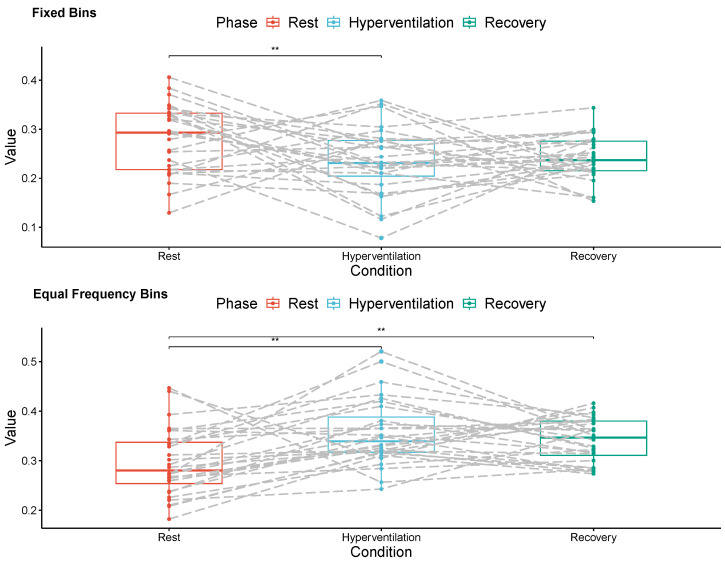
Mean conditional transfer entropy values for all volunteers, estimated from right hemisphere MCAv signals recorded during three respiratory phases (Rest, Hyperventilation, Recovery). The signals were discretized into 12 symbols using fixed bins (**top**) and equal frequency bins (**bottom**). The symbol ** indicates a significant difference.

Figure 3 and Figure 4 show paired mean values of conditional transfer entropy for each subject in the left and right hemispheres, respectively, during the three respiratory phases (Rest, Hyperventilation, and Recovery).

Table 1 details mean ± standard deviations values of conditional transfer entropy obtained from the left and right hemisphere MCAv signals during each phase of the respiratory exercise.

Averaged measures of conditional transfer entropy on the left hemisphere were significantly lower during normal breathing ( 0.295±0.066 ) than during both hyperventilation ( 0.363±0.056,p<0.001 , 22.0% increase) and the recovery phase ( 0.340±0.048,p=0.005 , 15.3% increase) when equal frequency bin discretization was employed. However, no significant differences were detected within this hemisphere using fixed bin discretization.

Within the right hemisphere, higher mean values of conditional transfer entropy gauged with equal frequency bin discretization were also observed during hyperventilation ( 0.356±0.067,p<0.001 , 20.3% increase) and recovery ( 0.341±0.044,p=0.007 , 15.2% increase) than in the resting phase ( 0.296±0.067 ). In this hemisphere, fixed bin discretization yielded higher mean values of conditional transfer entropy during the rest phase ( 0.280±0.070 ) than during hyperventilation ( 0.235±0.069,p=0.025 , 16.9% decrease).

## 4. Discussion

Both hypocapnia and hypercapnia have a profound impact on cerebral circulation, with significant changes in MCAv and moderate changes in BP [21]. Previous studies have often employed linear methods to assess MCAv responses to fluctuations in ETCO_2_, disregarding the influence of BP and the dynamic characteristics of this relationship. This may be due to technical difficulties in quantifying the contributions of these variables separately [22]. However such an approach can lead to misinterpretations [23].

There have been several attempts to measure the individual contributions of ETCO_2_ and BP to the observed fluctuations in MCAv signals (e.g., [8,21,23,24,25,26]). However, the nonlinear nature of the relationships between these variables has been considered less frequently (e.g., [26]). Understanding these nonlinearities is crucial, and may provide deeper insights into the physiological mechanisms at play.

Transfer entropy offers the possibility of surpassing linear methods and other bivariate methods, such as mutual information, by capturing the relation in terms of information flow between multiple variables. Moreover, the conditional version of transfer entropy is explored here as a simple tool to additionally quantify the individual information contribution of ETCO_2_ on MCAv fluctuations, excluding the effects of BP. This is highly relevant as transfer entropy, in common with many other measures of predictability, can be affected both by the presence of redundancy (input signals share features that are useful in predicting the output signal) and by the presence of synergy (the input signals together provide features that help predict the output signal and are not found when analyzed individually) causing information transferred from the inputs to the output to be underestimated or overestimated [27]. Conditional transfer entropy would not be affected in this way as redundant information is conditioned out while capturing any synergistic effects provided by the sources in combination [11] (p. 146).

Estimates of conditional transfer entropy with equal frequency bin discretization suggest the influence of blood CO_2_ levels on MCAv was more pronounced in hypercapnic (and semi-hypocapnic) conditions, compared to the poikilocapnic condition, which is consistent with the literature [23]. This superiority was observed in both brain hemispheres.

In addition, the same approach could detect a more significant influence of CO_2_ levels on MCAv during hyperventilation than during the recovery phase on the left hemisphere only, as might be expected if there are no delays in the return to normocapnia.

Measures of conditional transfer entropy gauged with fixed bin discretization only indicated a higher influence of CO_2_ during the rest phase compared to hyperventilation, which contradicts the expected results.

These findings indicate that equal frequency bin discretization yielded a more sensitive approach than fixed bin discretization. One plausible explanation for this discerning superiority may lie in the more intricate representation provided by the former. In equal frequency bin discretization, the signal is binned in a manner that ensures an equal number of values fall into each bin. As a result, the symbols assigned to each bin tend to appear with a similar frequency along the sequence representing the discretized signal (Figure 1), fostering a richer variety of patterns. Conversely, in fixed bin discretization, where bins have uniform sizes, symbols linked to bins closer to the median value appear much more frequently than those associated with more extreme amplitudes (Figure 3). Consequently, the observed patterns are less intricate.

It becomes clear that, at least in this application, conditional transfer entropy using equal frequency bin discretization detected the augmented influence of CO_2_ expected during hyperventilation, with the exception of the transition period to normal breathing on the left hemisphere. This result may be explained by either a slower recovery to the normocapnic state than the time period analyzed in this protocol or a lack of statistical power. More research is needed to elucidate this. Moreover, these positive results were not observed when the fixed bin discretization method was employed. It would be beneficial to explore other methods to discretize the signals, such as adaptive partitioning and kernel density estimation. However, it should be taken into consideration that these methods involve parameterization and demand substantial computational time in their calculations, unlike the methods applied in this study [5].

There are some limitations to this study. First, it cannot be guaranteed that volunteers, while controlling their breathing following a metronome tempo, are not also engaged in a cognitive task that may alter cerebral hemodynamics. Second, discrete probability functions were used for the calculation of transfer entropy as an approximation of continuous probability density functions. Finally, the approach was not assessed under hypercapnic conditions. For completeness, future studies should aim to undertake these measurements.

## 5. Conclusions

This study has demonstrated that conditional transfer entropy can effectively detect changes in the impact of CO_2_ levels on MCAv during poikilocapnic conditions, particularly in normocapnia and hypocapnia, while excluding the effects of blood pressure. This detection was achieved using the equal frequency bin discretization method. Moreover, the directionality of the change could be identified as the relevance of CO_2_ increased during hyperventilation. This study is considered a precedent for understanding the capabilities of transfer entropy in the study of cerebral hemodynamics. Future research is necessary to evaluate the proposed approach to study the participation of other variables of interest, such as BP and in hypercapnic conditions, on cerebral hemodynamics in both healthy subjects and patients with cerebrovascular disease.

## Figures and Tables

**Table 1:** Subject mean values of conditional transfer entropy. FBD: Fixed bin discretization, EFD: Equal frequency bin discretization. ^†^: significant difference with mean conditional transfer entropy at Rest.

Brain	Discretisation	Respiratory Exercise Phase		
Side	Method	Rest	Hyperventilation	Recovery
Right	FBD	0.280±0.070	$0.235\pm 0.069^{†}$	0.240±0.046
	EFD	0.296±0.067	$0.356\pm 0.067^{†}$	$0.341\pm 0.044^{†}$
Left	FBD	0.271±0.070	0.243±0.067	0.247±0.042
	EFD	0.295±0.066	$0.363\pm 0.056^{†}$	$0.340\pm 0.048^{†}$

## Data Availability

Available upon reasonable request to V.J.H.

## Abbreviations

The following abbreviations are used in this manuscript:

BP	Blood pressure
CO_2_	Carbon dioxide
EFD	Equal frequency bin discretization
ETCO_2_	End-tidal carbon dioxide
FBD	Fixed bin discretization
MCAv	Middle cerebral artery blood flow velocity
$T_{X\to Y}$	Transfer entropy from variable X to variable Y
$T_{X\to Y|Z}$	Conditional transfer entropy from variable X to variable Y, conditioned by the interaction of the variable Z

## References

[1] Payne S. (2016). Cerebral Autoregulation: Control of Blood Flow in the Brain.

[2] Gao J., Hu J., Tung W.W. (2012). Entropy measures for biological signal analyses. Nonlinear Dyn..

[3] Schreiber T. (2000). Measuring information transfer. Phys. Rev. Lett..

[4] Barnett L., Barrett A.B., Seth A.K. (2009). Granger causality and transfer entropy are equivalent for Gaussian variables. Phys. Rev. Lett..

[5] Lee J., Nemati S., Silva I., Edwards B.A., Butler J.P., Malhotra A. (2012). Transfer entropy estimation and directional coupling change detection in biomedical time series. Biomed. Eng. Online.

[6] Luo D., Pan W., Li Y., Feng K., Liu G. (2018). The interaction analysis between the sympathetic and parasympathetic systems in CHF by using transfer entropy method. Entropy.

[7] Harmah D.J., Li C., Li F., Liao Y., Wang J., Ayedh W.M., Bore J.C., Yao D., Dong W., Xu P. (2020). Measuring the non-linear directed information flow in schizophrenia by multivariate transfer entropy. Front. Comput. Neurosci..

[8] Ainslie P.N., Duffin J. (2009). Integration of cerebrovascular CO_2_ reactivity and chemoreflex control of breathing: Mechanisms of regulation, measurement, and interpretation. Am. J. Physiol. Regul. Integr. Comp. Physiol..

[9] Hanby M.F., Panerai R.B., Robinson T.G., Haunton V.J. (2017). Is cerebral vasomotor reactivity impaired in Parkinson disease?. Clin. Auton. Res..

[10] Jara J., Morales-Rojas C., Fernández-Muñoz J., Haunton V.J., Chacón M. (2021). Using complexity–entropy planes to detect Parkinson’s disease from short segments of haemodynamic signals. Physiol. Meas..

[11] Bossomaier T., Barnett L., Harr M., Lizier J.T. (2016). An Introduction to Transfer Entropy: Information Flow in Complex Systems.

[12] Stramaglia S., Cortes J.M., Marinazzo D. (2014). Synergy and redundancy in the Granger causal analysis of dynamical networks. New J. Phys..

[13] Lizier J.T., Prokopenko M., Zomaya A.Y. (2010). Information modification and particle collisions in distributed computation. Chaos Interdiscip. J. Nonlinear Sci..

[14] Montalto A., Faes L., Marinazzo D. (2014). MuTE: A MATLAB toolbox to compare established and novel estimators of the multivariate transfer entropy. PLoS ONE.

[15] Gourévitch B., Eggermont J.J. (2007). Evaluating information transfer between auditory cortical neurons. J. Neurophysiol..

[16] Duan P., Yang F., Chen T., Shah S.L. (2013). Direct causality detection via the transfer entropy approach. IEEE Trans. Control Syst. Technol..

[17] Shovon M.H.I., Nandagopal N., Vijayalakshmi R., Du J.T., Cocks B. (2017). Directed connectivity analysis of functional brain networks during cognitive activity using transfer entropy. Neural Process. Lett..

[18] Kale P., Acharya J.V., Acharya J., Subramanian T., Almekkawy M. Normalized transfer entropy as a tool to identify multisource functional epileptic networks. Proceedings of the 2018 40th Annual International Conference of the IEEE Engineering in Medicine and Biology Society (EMBC).

[19] Benedetto F., Mastroeni L., Quaresima G., Vellucci P. (2020). Does OVX affect WTI and Brent oil spot variance? Evidence from an entropy analysis. Energy Econ..

[20] Dash R., Paramguru R.L., Dash R. (2011). Comparative analysis of supervised and unsupervised discretization techniques. Int. J. Adv. Sci. Technol..

[21] Dumville J., Panerai R., Lennard N., Naylor A., Evans D. (1998). Can cerebrovascular reactivity be assessed without measuring blood pressure in patients with carotid artery disease?. Stroke.

[22] Panerai R.B., Evans D.H., Naylor A.R. (1999). Influence of arterial blood pressure on cerebrovascular reactivity. Stroke.

[23] Claassen J.A., Zhang R., Fu Q., Witkowski S., Levine B.D. (2007). Transcranial Doppler estimation of cerebral blood flow and cerebrovascular conductance during modified rebreathing. J. Appl. Physiol..

[24] Hetzel A., Braune S., Guschlbauer B., Dohms K. (1999). CO_2_ reactivity testing without blood pressure monitoring?. Stroke.

[25] Panerai R., Deverson S., Mahony P., Hayes P., Evans D. (1999). Effect of CO_2_ on dynamic cerebral autoregulation measurement. Physiol. Meas..

[26] Mitsis G.D., Poulin M.J., Robbins P.A., Marmarelis V.Z. (2004). Nonlinear modeling of the dynamic effects of arterial pressure and CO_2_ variations on cerebral blood flow in healthy humans. IEEE Trans. Biomed. Eng..

[27] Porta A., Bari V., De Maria B., Takahashi A.C., Guzzetti S., Colombo R., Catai A.M., Raimondi F., Faes L. (2017). Quantifying net synergy/redundancy of spontaneous variability regulation via predictability and transfer entropy decomposition frameworks. IEEE Trans. Biomed. Eng..

